# Why take the risk? Exploring the psychosocial determinants of floodwater driving

**DOI:** 10.3389/fpsyg.2022.913790

**Published:** 2022-07-19

**Authors:** Shauntelle Benjamin, Melissa Parsons, Deborah Apthorp, Amy D. Lykins

**Affiliations:** ^1^School of Psychology, University of New England, Armidale, NSW, Australia; ^2^Bushfire and Natural Hazards Cooperative Research Centre, Melbourne, VIC, Australia; ^3^School of Humanities, Arts, and Social Sciences, University of New England, Armidale, NSW, Australia; ^4^School of Computing, The Australian National University, Canberra, ACT, Australia

**Keywords:** personality traits, cultural worldviews, floodwater driving, risk-taking behavior, mood states

## Abstract

As anthropogenic climate change progresses, there is an increasing need for individuals to make appropriate decisions regarding their approach to extreme weather events. Natural hazards are involuntary risk environments (e.g., flooded roads); interaction with them cannot be avoided (i.e., a decision must be made about how to engage). While the psychological and sociocultural predictors of engagement with voluntary risks (i.e., risk situations that are sought out) are well-documented, less is known about the factors that predict engagement with involuntary risk environments. This exploratory study assessed whether mental health (depression, anxiety, and stress symptoms), personality traits, and cultural worldviews combine to predict engagement with involuntary risk, using the situation of floodwater driving. An Australian sample (N = 235) was assessed via questionnaire and scenario measures. Results were analyzed in a binomial logistic regression assessing which individual factors predicted decision-making in a proxy floodwater driving scenario. Agreeableness and gender were individually significant predictors of floodwater driving intention, and four factors (named “affect,” “progressiveness,” “insightfulness,” and “purposefulness”) were derived from an exploratory factor analysis using the variables of interest, though only two (“progressiveness” and “insightfulness”) predicted floodwater driving intention in an exploratory binomial logistic regression. The findings highlight the need for further research into the differences between voluntary and involuntary risk. The implication of cultural worldviews and personality traits in interaction with mental health indicators on risk situations is discussed.

## Introduction

The overwhelming majority of climate scientists agree that anthropogenic climate change is occurring (Doran and Zimmerman, 2009; Rosenberg et al., 2010; Powell, 2016), with remaining debates focused only on timing and degree: in other words, how fast humans will experience the consequences of rising world temperatures, and just how severe these will be. While there are a variety of predicted environmental effects of climate change (see IPCC, 2014 for review), the increased severity (and possibly frequency) of acute natural hazard events such as floods, wildfires, hurricanes, and other such extreme weather events represent a particular outcome of interest to health professionals, due to their likely impact on human health and wellbeing. These global changes, occurring in concert with a growing world population, suggest that even greater numbers of people are likely to face increasingly severe environmental threats going into the future. Consequently, understanding how individuals respond to environmental threats is crucial to mitigate future risk to human wellbeing associated with exposure to extreme weather events.

### The effects of natural hazard exposure

The varied and sometimes long-term mental health effects of natural hazard exposure have been well documented. Though people generally are resilient, and many do not develop mental illness following exposure to a traumatic event (Norris et al., 2009; Pietrzak et al., 2012), those that do can suffer significant impacts on their overall wellbeing and functioning. Reviews of the post-disaster mental health literature (see Norris et al., 2002; Goldmann and Galea, 2014) indicate that post-traumatic stress disorder is experienced by 30–40% of direct disaster victims, and major depressive disorder might have the highest post-disaster prevalence of all psychological disorders examined. There are some indications that disorders such as generalized anxiety disorder, panic disorder, and phobias might also increase in prevalence following disaster exposure, and many victims report non-specific psychological sequelae such as sleep disruption, feelings of grief and anxiety, and physical symptoms such as headaches, fatigue, abdominal pain, and shortness of breath (Goldmann and Galea, 2014). Norris et al. (2009) reported that following exposure to disaster, only 11% of the samples they reviewed experienced minimal or transient impairment, 51% evidenced moderate impairment, and 21 and 18%, respectively, showed severe or very severe impairment pointing to the development of one or more diagnosable psychological disorders. Typically, most persons experienced a peak of symptom presentation within the first year post-exposure that then improved over time. However, a significant minority of victims had symptoms that persisted for months or years, pointing to the ongoing nature of the psychological impact disaster exposure can precipitate.

A great deal is known about the psychological effects of natural hazard exposure, and the likely consequences for a substantial minority of those directly exposed to these events is predictable. However, in line with general clinical opinion as well as those of national psychological associations and research institutions (e.g., National Institute of Mental Health, World Health Organization, Australian Psychological Society), preventing mental illness is far preferable to working to manage and relieve it once it has developed. Recent reports from Australia indicate that mental health problems cost the economy over $220 billion annually (Australian Government Productivity Commission, 2020), an estimate that still does not account for the emotional and social costs associated with mental illness. Thus, for many important reasons, the ideal situation is to prevent the development of mental illness. It is clear that not all exposure to natural hazards is avoidable, but there is a diversity of behavioral responses when faced with a natural hazard, ranging from avoiding threats when risk is known to be high (e.g., evacuating during a bushfire or hurricane, not driving through flooded roads) to electing to engage with the hazard (e.g., staying to fight bushfires, sheltering in place during a hurricane, driving through a flooded road). Irrespective of other environmental factors, it is possible, and perhaps even likely, that core intrapersonal features and/or mental health states influence responses to engage with or avoid natural hazards. Further exploration of the interactions between social and psychological influencers and their effect on risk-taking behavior is urgently needed if we are to prepare for the increasing severity and frequency of natural hazard events.

### Individual factors associated with risk-taking

With respect to who engages in risky behavior when faced with an environmental threat, there is a large body of research identifying the demographic characteristics and environmental factors that are associated with risky decision-making. Taking floodwater driving as an example, men (Haynes et al., 2016), those who are driving a 4-wheel-drive automobile (Gissing et al., 2015), and persons who are following the instructions of others (Hamilton et al., 2020) are most likely to attempt to drive through flooded roadways. Previous experience with having driven through floodwaters is also associated with doing so again (Taylor and Haynes, 2019; Ahmed et al., 2020). Despite targeted government advertising highlighting the dangers associated with floodwater driving, fatalities have been increasing in Australia (Gissing et al., 2015), with more than half of unintentional flood-related drowning deaths resulting from floodwater driving (Pearson and Hamilton, 2014; Australian Water Safety Council, 2016).

Risk-taking and personality traits in driving situations have been queried in other areas, such as speeding and drink driving (e.g., Tinella et al., 2021), however, there may be implicit differences between those who engage in risky behavior unprompted by external factors and those presented with inherently risky situations and choose to then engage in risk-taking behavior. While personality traits have been noted as a distal influencer on proximal driving factors such as driving skills, the relationship between personality traits and driving behavior is complex, given the variations in context and number of variables that influence behavior in this setting (Tinella et al., 2021). Tinella et al. (2021) queried whether demographic information was a distraction from underlying personality traits, however, found that information such as age, gender and experience or education are critical influencers impacting driver behavior in areas such as awareness, visual acuity, and reaction speed. Demographically, the people who engage in these behaviors are generally consistent with the findings described above. Moreover, in situations such as speeding and traffic violations, increased experience has been found to be consistent with increased violations, potentially due to a sense that they understand the road and its hazards better than less experienced drivers (Trapsilawati et al., 2021). Trapsilawati et al. (2021) also found that perception or awareness of what consequences the individual could face influence driver behavior. Married drivers with children and older drivers reported increased awareness of legal road requirements, while single and younger drivers were less responsive to these mechanisms, suggesting that risk awareness and aversion may be related to awareness of potential material loss. As demographics such as age and gender are intrinsically linked to sociocultural structures and expectations (Figner and Weber, 2011), risk-taking behavior is necessarily going to be influenced by a combination of biological, psychological and sociocultural influencers.

Despite the information known about who is most likely to engage in risky behavior when faced with an environmental threat such as a flooded road, and under what social and environmental circumstances, we know significantly less about why and how these decisions are made. Are there intrapersonal characteristics that place a person at greater risk of engaging with an environmental threat? If such characteristics can be found, this information could be used to identify individuals with a higher propensity for engaging with these risky situations. Interventions may then be developed to mitigate individuals’ innate proclivities for environmental risk-taking, thus preventing the potential development of subsequent mental health difficulties due to direct natural hazard exposure.

Given the lack of literature about the intrapersonal characteristics that influence engagement with an environmental threat, we turned to the literature on intrinsic psychological drivers (both clinically-relevant and socially-based) of risk-taking behavior in general. The clinically-relevant literature points to two major areas of interest: the effects of mental states such as anxiety and depression on decision-making, and the effects of personality factors (particularly in their extreme forms, potentially indicating personality pathology) on risk engagement.

#### Mood states and risk-taking

Research findings about the relationship between negative mood and/or affect and risk-taking are somewhat mixed. Generally, negative mood or affect, and depression specifically, show a positive correlation with risk-taking, such that when negative mood is higher, the propensity for engaging in risky behavior is also higher. However, this pattern sometimes varies depending on how the person has come to experience his or her negative mood (i.e., pre-existing depression vs. sad mood induction) and what type of risk has been assessed. Samples of persons self-reporting or actually diagnosed with clinical depression have shown higher propensities for sexual (Alvy et al., 2011; Wilson et al., 2014; Millar et al., 2017), financial (Blaszczynski and McConaghy, 1989; Bayer et al., 2019), and general (Desrichard and Denarie, 2005) risk-taking. However, Cobb-Clarke et al. (2019) found a higher propensity for health but not general risk-taking in their depressed participants when assessed against their non-depressed participants. Interestingly, mood induction studies have largely found the opposite pattern, with greater negative mood increasing risk aversion (Yuen and Lee, 2003; Chou et al., 2007), though Mittal and Ross (1998) found that participants who experienced a negative mood induction showed higher levels of risk-taking than those in the positive mood induction condition. Herman et al. (2018) found that negative emotions were associated with increased impulsivity, while positive affect was related to increased risk-taking. The authors in this case noted that negative affect, and therefore impulsivity can, but may not necessarily, lead to increased risk-taking. Overall, these results suggest that while persons experiencing a temporary negative mood may become more risk averse, those experiencing a persistently negative mood are likely at higher risk of engaging in risky behaviors, potentially due to reduced impulse control or as a coping mechanism used in effort to relieve the negative mood state.

In contrast, the results of studies investigating how anxiety and stress influence risk-taking have mostly shown the opposite pattern. Maner et al. (2007) reported that participants with higher dispositional anxiety showed greater risk avoidance than non-anxious participants, as did Giorgetta et al. (2012) for participants with generalized anxiety or panic disorder. Giongetta et al. interpreted their results as indicative of a hypersensitivity to potential threats and pessimism associated with future events resulting in lower risk-taking engagement. However, Renier et al. (2016) reported that adolescents with higher social anxiety were more susceptible to risk-taking than those with lower social anxiety, suggesting that anxiety involving interpersonal interactions may place some people (at least, adolescents) at greater risk of engaging in risky behavior. Lastly, von Helversen and Rieskamp (2019) showed no main effect of experiencing a stress induction on financial risk-taking; however, participants who reported higher negative affect prior to the stress induction did show an increase in risky decision-making that did not occur in the non-stress-induction condition, indicating important interactions between negative mood and stress and consequent behavioral outcomes.

#### Personality and risk-taking

In an effort to understand whether more enduring intrapersonal characteristics might put an individual at higher risk for engaging in risk-taking behavior, many researchers have turned to examining the relationships between personality traits and risk-taking. Though there have been a number of models of personality structure over the past decades, the model with the greatest amount of empirical support is the Five Factor Model, or the “Big Five” (McCrae and John, 1992). This model proposes that a person’s overall “personality” is constructed by the degree to which they experience the core features of openness to experience, conscientiousness, extraversion, agreeableness, and neuroticism (i.e., emotional instability; Costa et al., 2019). Test-retest intervals of 6–15 years have shown little change in the endorsement of the Big Five personality traits over time (Terracciano et al., 2006), providing support for their enduring nature. Furthermore, Big Five personality traits have been found to predict a wide range of positive and negative outcomes across the lifespan, and more recently have begun to be linked to diagnosable personality disorders (DSM-5; American Psychiatric Association, 2013).

Though the reported prevalence of personality disorders in Australia is relatively low at 6.5% (Jackson and Burgess, 2000) with a skew toward men that likely reflects the direct prevalence rate (in which individuals have been provided with a diagnosis), it is believed that the provisional prevalence rate (the rate at which a diagnosis might be provisionally provided) is likely to be higher. Furthermore, it is likely that even higher percentages of the overall population experience subclinical levels of one or more of the symptoms of a personality disorder—levels not reaching diagnostic thresholds but still impacting overall wellbeing and functioning. Research on maladaptive levels of the Big Five personality traits have shown extreme conscientiousness and its relationship to compulsivity to be associated with obsessive-compulsive personality disorder (Carvalho et al., 2019a), high levels of openness related to magical thinking have been associated with aspects of psychosis (Widiger and Crego, 2019), extreme levels of agreeableness associated with subservience as seen in dependent personality disorder (Carvalho et al., 2019b), a lack of neuroticism relating to fearlessness (Friedman, 2019), and high levels of extraversion resulting in dominance (Watson et al., 2019) associated with aspects of narcissistic personality disorder.

Through the construct of personality traits, it is possible to see how enduring maladaptive cognition and affect can result in risky decision-making. In a brief review of the literature on the Big Five personality traits and risk-taking using different measures, assessing different age groups, and investigating a variety of different types of risky behaviors, some clear patterns emerge. One of the most consistent findings is the relationship between higher extraversion and greater propensity for risk-taking, which has been found from early (McGhee et al., 2012) to middle (Gullone and Moore, 2000) adolescence through to adulthood (Nicholson et al., 2005; Tok, 2011; Thompson and Prendergast, 2015; Czerwonka, 2019) across a range of different potentially risky situations. Higher openness to experience (Nicholson et al., 2005; Tok, 2011; McGhee et al., 2012) and lower conscientiousness (Gullone and Moore, 2000; Tok, 2011; McGhee et al., 2012; Thompson and Prendergast, 2015; Czerwonka, 2019) have also been associated with higher propensities for risk-taking.

The findings for agreeableness and neuroticism are mixed, and these traits do not regularly account for a significant amount of variance in risk-taking propensity. Risk-taking adolescents scored higher on agreeableness (Gullone and Moore, 2000), possibly suggesting a greater susceptibility to risk-tasking associated with peer pressure, but no other studies reviewed indicated that agreeableness significantly contributed to risk-taking in either direction. Lower neuroticism was associated with greater risk-taking in sports (Tok, 2011) and across a number of different domains (e.g., recreation, health, career, finance, safety) in the Nicholson et al. (2005) study; however, higher neuroticism was associated with greater impulse buying (Thompson and Prendergast, 2015) and more risk-taking in parkour enthusiasts (Merritt and Tharp, 2013) in other studies. Overall, research investigating the contribution of Big Five personality factors to risk-taking suggest that these traits together account for a large percentage of variance present in risk-taking behavior, reinforcing their relevance in examining various types of risk-taking behavior.

#### Cultural worldviews and risk-taking

Less clinically relevant but still pertinent to risk-taking behavior is the relationship between sociocultural worldviews and the perception of risk. The cultural theory of risk (Wildavsky and Dake, 1990) proposes a 2 × 2 matrix of culturally-based risk analysis that both reflects and reinforces one’s own preferences for social organization and culturally-derived ways of life. According to Douglas and Wildvasky (1982), the “grid” axis reflects preferences for social stratification and roles of authority; as such, high grid scores reflect beliefs in the immutability of status (i.e., hierarchism), whereas low grid scores reflect beliefs that no individual should be excluded from social roles based on their demographic characteristics (i.e., egalitarianism). The “group” axis reflects a person’s preferences for individualism vs. communitarianism; persons higher on the group axis prefer systems that foster self-reliance and competition (i.e., individualism), whereas persons lower on the group axis prefer social structures that foster strong social bonds and cooperation (i.e., communitarianism).

An oft-mentioned example of the differences across the axes is the focus on environmental risk perception (Kahan, 2012). With respect to environmental threat, people high on the group axis (i.e., high on individualism) could be expected to dismiss environmental concerns due to the threat it introduces to their way of life, while those low on group characteristics would likely be disdainful of the individualistic behaviors involved in dismissing environmental concern, viewing the focus on industry as selfish or uncompromising. Comparatively, those high on hierarchical endorsement (i.e., high grid characteristics) can be predicted to dismiss environmental concerns due to the implicit judgment of authority that occurs in such discussions, while those low on grid characteristics (i.e., high egalitarianism) are more likely to question the judgment of those across rank lines, because such markers are deemed to be unjust.

#### Voluntary vs involuntary risk situations

The preceding information briefly reviews what is known about psychological and sociocultural factors associated with risk-taking in general. However, there may be reason to examine whether the same cognitive or behavioral patterns occur when engaged by an involuntary risk situation that confronts an individual (e.g., flooded roads) compared to when a risk situation is voluntary, or approached (e.g., abseiling). The majority of the cognitive and behavioral studies reviewed have examined mental states and personality factors that predict engagement of risky behaviors in voluntary situations that a person seeks out, such as sexual behavior, alcohol and drug use, gambling, extreme sports, and speeding while driving, and thus may serve as a proxy assessment of propensities for sensation seeking (Skeel et al., 2007). In the case of environmentally threatening situations, however, most persons who experience natural hazards are involuntarily confronted with them without their consent (Rehm et al., 2014). As such, we suggest that there are two dimensions to the voluntariness of a situation when engaging in risk-taking behavior. The first is whether the individual can decide whether to be exposed to the risk situation or environment. This refers to the seeking out of risk situations (e.g., drug seeking behavior), but also applies to situations that could be avoided if the individual leaves the environment (e.g., stockbroking). The second is the decision of how to engage with that risk. In our conceptualization, the bulk of research assesses how individuals behave in response to this decision. In the case of natural hazards, there is no ability to control whether there will be exposure to the risk situation. The only decision available to individuals is how to engage. In this way, predictors of risky environmental decision-making may be qualitatively and quantitatively different from what we know about risk-taking in general. Given natural hazards are involuntarily experienced and not sought out, there may not be a typical profile of a person who is faced with them. So, what does the average person do when faced with an environmentally-threatening situation, and what clinically-relevant and sociocultural characteristics drive these decisions?

#### The current study

In the current study, we aimed to address this gap in the literature by investigating the relative contribution of clinically relevant psychological and sociocultural characteristics to risk-taking behavior in a proxy situation of involuntary environmental threat, using floodwater driving as a test case. Due to the relative lack of information on psychological predictors of risky decision-making in natural hazard events, or the potential interactions amongst personality traits, floodwater driving and mental health indicators, for this exploratory study we relied on the results of studies that examined individual drivers of risk-taking in general to develop hypotheses. We focused on indicators of mental health and mental states (e.g., depression, anxiety, and stress symptoms) and personality factors associated with the Big Five (e.g., neuroticism, extraversion, openness to new experiences, agreeableness, and conscientiousness). We also assessed the contribution of sociocultural worldviews (e.g., hierarchism, egalitarianism, individualism, communitarianism) to this decision-making process, as these variables have been shown to be associated with environmental risk perceptions (Xue et al., 2014).

To assess risk-taking behavior in a physically safe situation, we developed a proxy floodwater driving setting administered using a Situational Judgement Test (Ng and Rayner, 2010). Participants were presented with a situation in which they have approached a flooded roadway in their own cars and were asked what they would do in this situation: drive through or find a different route.

Based on the extant psychological risk-taking literature, and the literature about floodwater driving, we hypothesized that participants who are more likely to drive through flooded roadways than to turn around would evidence:

1. Higher levels of depressive symptoms;

2. Lower levels of anxiety and stress;

3. Lower conscientiousness;

4. Higher extraversion;

5. Higher openness;

6. Higher levels of group cultural worldview;

7. Lower levels of grid cultural worldview;

8. Identify as male;

9. Be younger rather than older adults.

## Method

### Participants

Overall, 337 adults residing in Australia engaged with the online survey in October 2018. Data were removed for persons who did not complete all aspects of the survey, for those who completed the survey in less than half the median completion time, for those whose qualitative responses indicated that they had not completed the study in earnest, and for those who reported having had no first-hand driving experience. The final sample comprised 235 adults (M_age_ = 47.46, SD = 18.05, range = 18–82), of whom 60% (n = 141) identified as male and 40% (n = 94) identified as female. Regarding education, 60% of participants reported their highest educational attainment as a trade certificate or higher. The Australian Bureau of Statistics reports that the median age for capital cities is 36 years, which is younger than the median for the rest of Australia (41.2; Australian Bureau of Statistics, 2020a,b). Both are younger than the reported median age of our sample (49 years of age). Our sample reported lower weekly income on average (approximately AUD$1,153) than the national mean (AUD$2,242) for 2017. Seventy per cent of the sample reported more than 10 years of driving experience, and consistent with previous research, 48.1% reported having driven through floodwater in the past.

Qualtrics (Provo, Utah) was hired to recruit participants for this study. They were responsible for all aspects of advertising and recruitment to the study via their online survey panels. Participants received minor incentives for their participation, as determined and managed by Qualtrics. This study received approval from the University of New England’s Human Research Ethics Committee prior to participant recruitment.

### Measures

#### International personality item pool

The International Personality Item Pool (IPIP; Maples et al., 2014) is a public domain collection of items that can be used to assess personality traits. Participants were given a series of statements and asked to respond to them by describing themselves as they generally are, with response options ranging from 1 = “very inaccurate” to 5 = “very accurate,” with higher scores on the subscales indicating higher levels of that particular trait. For the present study, the Maples et al. (2014) 120-item measure was used to assess personality traits and symptomology consistent with the NEO Five-Factor Personality Inventory (Costa and McCrae, 1992). This 120-item measure has shown good convergent validity with the NEO-PI-R across each trait, with correlations ranging from 0.88 to 0.91 (Maples et al., 2014). In the present sample, assessments of internal consistency resulted in Cronbach’s alphas of 0.68 for Conscientiousness (questionable), 0.74 for Openness (acceptable), 0.79 for Neuroticism (acceptable), 0.80 for Agreeableness (good), and 0.88 for Extraversion (good).

#### Depression, anxiety, and stress scale

The Depression, Anxiety and Stress Scale (DASS-21; Lovibond and Lovibond, 1995) was administered to assess general mental health and wellbeing over the preceding week. Participants were asked to respond to 21 items such as, “I found it hard to wind down,” with response options ranging from 0 = “never” to 3 = “almost always.” Scale scores ranged from 0 to 21, with higher scores on the DASS-21 subscales indicating higher levels of depression, anxiety, and stress symptoms. The DASS-21 has been shown to have strong convergent and discriminant validity, as well as good internal consistency, with Cronbach’s alphas of 0.88 (depression), 0.82 (anxiety), and 0.90 (stress; Henry and Crawford, 2005). Cronbach’s alphas for the current sample were 0.95 for depression (excellent), 0.89 for anxiety (good), and 0.92 for stress (excellent).

#### Cultural cognition worldview scale

The short version of the Cultural Cognition Worldview Scale (CCWS; Kahan et al., 2011) was used to assess participants’ endorsements of cultural worldviews across two orthogonal axes of grid and group (Kahan et al., 2007). Participants were asked to respond to 12 items such as, “The government should stop telling people how to live their lives,” with response options ranging from 1 = “strongly disagree” to 7 = “strongly agree.” Higher scores on these two axes indicate greater endorsements of hierarchy or individualism, and thus lower endorsements of egalitarianism and communitarianism. The CCWS was designed and validated in the United States and has shown good internal consistency in American samples, with Cronbach’s alphas of 0.81 and 0.87 (Kahan et al., 2011). However, the internal consistency of these scales was questionable to acceptable in the current Australian sample, with Cronbach’s alphas of 0.61 and 0.76.

#### Situational judgement test

The participant’s proclivity to enter a flooded road while driving was assessed without endangering them using a Situational Judgement Test (SJT; Ng and Rayner, 2010). Participants were provided with a vignette to assess their behavioral responses to a flooded road while driving a car. The scenario read:

It has been raining heavily and there has been a rise in the water level of the local river. You are driving during the daytime. You come to a point where the road is flooded. You can see the water is flowing downstream across the road, but there is no indication of whether it will continue to rise, fall, or stay the same. You note that there is a white flood marker pole sticking out of the water at the side of the crossing. It shows a water depth of 20 cm.

The SJT was designed to provide a baseline scenario for what a person would do when presented with a flooded road under ideal circumstances. In this scenario, visibility was good, no one was in the car putting pressure on the participant to cross, and no information was provided indicating that the participant had an urgent reason to cross the road (e.g., to pick up a child)—all variables that have been shown to increase floodwater driving propensities in previous research (Wright et al., 2015). The depth of 20 cm was selected because at this depth, water will reach the underside of most cars, causing a potential floating hazard (Pearson and Hamilton, 2014). Previous research also has indicated that 20 cm is conceptualized as a low but still potentially risky scenario (Hamilton et al., 2016). After reading the scenario, participants were provided with four response options and asked to rank them in the order in which they would undertake each action. The response options were: “Continue in the direction I was going,” “Stop and assess the situation,” “Find another route,” and “Something else.”

### Procedure

Participants clicked on a link to the online survey through Qualtrics, where they were provided with information about the study. After providing consent, participants first answered the demographics questions. They were then presented with measures in the following order: (1) CCWS; (2) the DASS-21; (3) the IPIP; and (4) the Situational Judgement Test. Participants were then thanked for their time and their responses were recorded.

### Statistical analyses

This study utilized a correlational design to examine the individual psychological predictors impacting risk-taking behavior in an environmentally risky situation. To test our hypotheses, all data were analyzed using Jamovi computer software (The jamovi Project, 2022), except for factor scores, which were calculated using R (R Core Team, 2014) and the psych package (Revelle, 2021). To assess the determinants of floodwater driving in the Situational Judgement Test, we used binomial logistic regression to predict participant behaviors around floodwater driving using psychological and sociocultural factors. Though we had a priori hypotheses about which factors we expected to influence decision-making, we included all 10 variables of interest (i.e., depression, anxiety, stress, hierarchical-egalitarianism/grid scale, individualism-communitarianism/group scale, neuroticism, extraversion, openness, agreeableness, and conscientiousness) as well as two of the demographic variables (i.e., age and sex) that have been shown to be related to risk-taking in previous research. Additionally, we used exploratory factor analysis to discover whether latent constructs might underpin the decision-making process. We chose this method because the factors influencing engagement with involuntary risk may differ from those reported in the literature about voluntary risk, and so an exploratory approach was deemed appropriate. Finally, we ran an exploratory binomial logistic regression to assess which factors (among those derived from the factor analysis) could predict reported floodwater driving intentions.

Participant response decision options formed four categories reflective of the participant’s decision-making process: (1) drive straight through, (2) assess, then drive through, (3) assess, then take a different route, and (4) take a different route immediately, with a fifth option, “something else” to allow participants to qualify responses. These categories reflected the point at which the participant stopped engaging with the flooded road because they had either driven through it or turned around, irrespective of whether that happened as their first decision or second. To analyze the data, we collapsed the responses into a binary: individuals who drove through (drive straight through, or assess then drive straight through) and individuals who did not (take a different route immediately, or assess then take a different route).

## Results

Most participants (66.8%) reported the intention to stop and assess the situation prior to deciding; however, 21% of those who made the decision to assess then also reported the intention to drive through floodwater regardless. Correlations among the variables are reported in Table 1, and results of the logistic regression are reported in Table 2. While we asked participants to indicate their driving experience (in years), we found that this was highly correlated with age (r(233) = 0.84, p < 0.001). As such, we deemed that controlling for age was sufficient while avoiding multicollinearity as a result of including this demographic alongside.

**TABLE 1:** Correlation matrix of the variables of interest.

	1	2	3	4	5	6	7	8	9	10	11
1. Age	–										
2. Gender	−0.33***	–									
3. Group - CWV	0.04	−0.14*	–								
4. Grid - CWV	0.17*	−0.30***	0.40***	–							
5. Neuroticism - IPIP	−0.41***	0.30***	−0.20**	−0.20**	–						
6. Extraversion - IPIP	–0.07	–0.02	–0.11	–0.04	−0.29***	−−					
7. Openness - IPIP	−0.22***	0.20**	−0.24***	−0.42***	0.12	0.27***	–				
8. Agreeableness - IPIP	0.24***	0.14*	−0.14*	−0.43***	−0.16*	0.11	0.28***	–			
9. Conscientiousness - IPIP	0.29***	0.01	0.07	–0.01	−0.51***	0.35***	0.04	0.44***	–		
10. Depression - DASS	−0.27***	0.09	0.01	–0.06	0.68*	−0.31***	0.03	−0.15*	−0.33***	–	
11. Anxiety - DASS	−0.32***	0.07	0.03	0.00	0.61	−0.14*	–0.03	−0.25***	−0.36***	0.79***	–
12. Stress - DASS	−0.36***	0.15*	–0.03	–0.07	0.68	–0.12	0.11	−0.17***	−0.29***	0.84***	0.83***

*p < 0.05, **p < 0.01, ***p < 0.001.

**TABLE 2:** Binomial logistic regression assessing which variables predict floodwater driving.

			95% Confidence interval		
Predictor	b	SE	Lower	Odds ratio	Upper
Intercept	3.61	2.77	0.16	36.80	8411.14
Age	0.01	0.01	0.99	1.01	1.03
Depression	–0.04	0.05	0.86	0.96	1.06
Anxiety	0.10	0.06	0.98	1.11	1.25
Stress	0.02	0.07	0.90	1.02	1.16
Neuroticism – IPIP	–0.03	0.02	0.95	0.98	1.01
Extraversion – IPIP	–0.01	0.01	0.96	0.99	1.01
Openness – IPIP	–0.03	0.02	0.94	0.97	1.00
Agreeableness – IPIP	0.04*	0.02	1.01	1.04	1.08
Conscientiousness – IPIP	–0.01	0.02	0.96	0.99	1.02
Female – Male	0.91*	0.37	1.21	2.47	5.07
Group – CWV	–0.05	0.04	0.89	0.95	1.03
Grid – CWV	–0.04	0.03	0.91	0.96	1.01

Estimates represent the log odds of “Different route” vs. “Drove through.” **R^2^** = 0.11 (McFadden), 0.13 (Cox-Snell), 0.18 (Nagelkerke). Model χ**^2^**(12) = 31.8, p = 0.0001. *p < 0.05.

### Binomial logistic regression

Following assessment of the Akaike Information Criterion (AIC = 290) and analysis of McFadden’s, Cox and Snell and Nagelkerke goodness of fit tests (reported in Table 1), we conducted a binomial logistic regression on the 12 variables. As noted in Table 2, the only statistically significant predictors of intending to take a different route were agreeableness (*b* = 0.04,Waldχ^2^(1) = 6.66 p = 0.010) and gender (*b* = 0.91,Waldχ^2^(1) = 6.36,p = 0.012). At higher levels of agreeableness, participants were more likely to report the intention to take a different route. Females were more likely to report taking a different route than males.

### Additional analyses

#### Factor analysis

Given the high correlations between many of the variables, and the fact that, individually, most of our variables of interest were not directly associated with our behavior measure, we conducted an exploratory factor analysis to assess: (1) whether there were underlying, unidentified variables that clustered together amongst the 12 variables of interest, and (2) whether these factors (should they exist) better predicted the results of the SJT responses than the variables did individually. The exploratory factory analysis was conducted using principal axis factoring and orthogonal rotation (varimax) to assess whether underlying constructs existed among these 12 variables.

Sampling adequacy was “middling” (KMO = 0.75) as described using the standard set by Kaiser and Rice (1974). Initial eigenvalues and the scree plot both indicated that a 4-factor solution was appropriate (see Table 3). The first factor, which we named “affect,” explained 25.10% of variance within the factor and was comprised of the measure items of state depression, anxiety, stress and neuroticism. The second factor, named “progressiveness,” explained 14.24% of factor variance and was comprised of positively loaded neuroticism, gender, openness (to experience), and agreeableness, while age, group and grid cultural worldviews loaded negatively. As high scores on the cultural worldview scales indicate increasingly hierarchical and individualized cultural worldviews, a negative loading is indicative of the opposite constructs (egalitarianism and communitarianism, respectively), while age indicates younger individuals. The third factor, which we named “insightfulness,” comprised negatively loaded neuroticism and positively loaded agreeableness, conscientiousness, and age, explaining 10.90% of variance. The fourth factor, named “purposefulness” (after Witt, 2002) comprised positively loaded conscientiousness and extraversion, explaining 8.10% of variance.

**TABLE 3:** Factor analysis showing latent constructs between variables of interest.

	Factor				
		
	“Affect”	“Progressiveness”	“Insightfulness”	“Purposefulness”	Communalities
DASS – stress	0.93				0.10
DASS – depression	0.90				0.14
DASS – anxiety	0.86				0.22
IPIP – neuroticism	0.64	0.36			0.25
CWV – grid		–0.77			0.38
IPIP – openness		0.55			0.63
CWV – group		–0.45	–0.35		0.80
Sex		0.42			0.80
IPIP – agreeableness		0.42	0.73		0.28
IPIP – conscientiousness			0.58	0.36	0.47
Age		–0.32	0.46		0.58
IPIP – extraversion				0.78	0.35

“Principal axis factoring” extraction method was used in combination with a “varimax” rotation.

#### Binomial regression of factor scores

A binomial logistic regression using the factor scores derived from the original dependent variables reveals that only two of the factors, “progressiveness” (*b* = 0.33,Waldχ^2^(2) = 13.4 p = 0.046) and “insightfulness” (*b* = 0.49,Waldχ^2^(2) = 13.4 p = 0.005) significantly predicted reported decision-making. Further, these latent variables more accurately predicted the intention to take a different route (86.6%), rather than predicting the intention to drive through floodwater (19.3%). Results of this binomial regression are reported in Table 4.

**TABLE 4:** Logistic regression to assess the predictive ability of identified factors.

			95% Confidence interval		
Predictor	b	SE	Lower	Odds ratio	Upper
Intercept	0.57**	0.14	0.29	1.77	0.86
Factor 2 – “Progressiveness”	0.33*	0.16	0.005	1.39	0.65
Factor 3 – “Insightfulness”	0.50**	0.17	0.15	1.65	0.82
Factor 1 – “Affect”	3.40e-4	0.15	–0.29	1.00	0.29
Factor – 4 “Purposefulness”	–0.21	0.18	–0.56	0.81	0.13

Estimates represent the log odds of “Different route” vs. “Drove through”. **R^2^** = 0.05 (McFadden), 0.06 (Cox-Snell), 0.08 (Nagelkerke). Model χ**^2^**(2) = 13.4, p = 0.001. *p < 0.05, **p = 0.005, *p < 0.001.

## Discussion

In this study, we aimed to establish whether state mental health and affect, trait personality, and sociocultural characteristics predicted behavioral decision-making in an involuntary risk-taking situation. Trait agreeableness and gender identification were the only significant individual predictors of decision-making in the original regression analysis. Though we only found support for one of our original hypotheses, results of a binomial regression analysis using the factors revealed in an exploratory factor analysis indicated that the intention to avoid floodwater driving is likely to be impacted by two constructs that we have named “progressiveness” and “insightfulness.”

### Outcome of hypotheses

Of the psychosocial variables we used to predict risk-taking behavior (i.e., mental health indicators, cultural worldview and personality traits), only gender and trait agreeableness were significant individual predictors of decision-making. While our findings regarding gender and risk-taking were consistent with existing literature (Kahan et al., 2007; Hamilton et al., 2020), the fact that our other hypotheses were not confirmed is surprising, as negative affect has been shown to increase or decrease risk-taking proclivities in several studies (Malouff et al., 2005; Wise et al., 2015), and extraversion has been largely implicated as a predictor of other risk-taking activities (e.g., Breivik et al., 2019; Zhang et al., 2020). Additionally, in applying the cultural theory of risk to risk-perception associated with oil and gas development, McEvoy et al. (2017) found that individuals who aligned more with an increased individualistic worldview (high group cultural worldview) were more likely to express low risk perception when faced with an objectively risky situation. Given these differences are so well documented, our initial findings were somewhat unexpected.

### Exploratory factor analysis

Due to the lack of individual predictors, we ran an exploratory factor analysis and a further binomial logistic regression using the factor scores to assess whether groupings of variables better predicted risk-taking behavior than those variables on their own. We reduced our twelve initial predictors down to four latent variables, which we named “progressiveness” (more female, with positively loaded neuroticism, openness to experience, and agreeableness; negatively loaded age, group and grid cultural worldviews), “insightfulness” (negatively loaded neuroticism and positively loaded agreeableness, conscientiousness, and age), “affect” (state depression, anxiety, stress and neuroticism), and “purposefulness” (positively loaded conscientiousness and extraversion).

Of the four latent variables, two of them (“progressiveness” and “insightfulness”) significantly predicted an aversion to floodwater driving behavior in our sample. The latent variable of affect, combining neuroticism with mental health indicators, was to be expected, as neuroticism is largely conceptualized as a trait describing (the lack of) emotional stability (Widiger and Oltmanns, 2017). However, both positive and negative valences of neuroticism were predictive of risk-aversion within the constructs of “progressiveness” and “insightfulness.” As neuroticism is largely perceived to be a negative trait with broadly poor health outcomes (Widiger and Oltmanns, 2017), the possibility that the interaction of increased neuroticism with other traits such as openness to experience and agreeableness may provide protection against more extreme trait expression is notable.

Though we did not hypothesize an impact of agreeableness on floodwater driving, it should not be surprising that those individuals who indicated they would not drive through floodwater are also those more likely to report increased openness to experience and low hierarchism. Hierarchism is defined within this context as deference to authority (Kahan et al., 2011), however, increased hierarchism does not identify *in which* authority the individual places their trust. Those exhibiting increased openness to experience, and increased agreeableness, therefore may, in their rejection of authority, be seen to be more likely to consider their own ability to navigate the risk situation before choosing how to engage with it. This possibility is interesting given the fact that increased agreeableness has been associated with selflessness, subservience, and suggestibility (Widiger et al., 2012).

### The influence of voluntariness

The influence of agreeableness in voluntary risk situations may lend further credence to a potential difference between voluntary (approached) and involuntary (unavoidable) risk situations. Increased agreeableness conceptually aligns within both the “progressive” and “insightful” factors, both of which we found to be significantly predictive of floodwater driving aversion in our sample. As agreeableness can be conceptually viewed as collaborative, considerate, sociable, and altruistic (Graziano and Eisenberg, 1997), involuntary situations may lead such individuals to consider the macro effect of their risk-taking behavior rather than their own motivations. When compared to the sociability of extraversion, it is possible that the two traits work in opposition across involuntary and voluntary risk-taking, such that increased agreeableness fulfills a similar psychosocial function in involuntary situations that extraversion fulfills in voluntary situations. Specifically, it is possible that increased extraversion and low agreeableness (two “leader traits”; Aichholzer and Willmann, 2020) which are recognized as socially desirable in voluntary situations, become ineffective in involuntary risk situations, thus leaving those with increased agreeableness, conscientiousness, and openness to experience to find safer solutions to navigate undesirable environments. As such, both sets of traits would be required in a functioning community to navigate both emergency risk situations and risks that may provide community enhancement.

### Implications and limitations

Environmental threat is currently mitigated through several different means, including education programs (McNeill et al., 2016), legal regulations such as fines for passing barriers or engaging in risky behavior (Ahmed et al., 2018), and early warning systems and signage (Franklin et al., 2014). There is a limited understanding of the behavioral determinants around environmental risk-taking behavior, and as noted above, given reports that floodwater driving is increasing (Taylor et al., 2019), one positive finding is that most (66.8%) individuals stopped to assess the situation prior to making a decision, although 21% of those who stopped to assess the situation then made the decision to continue through despite this initial hesitation. As behavioral change is a complex endeavor, the present study provides some insight into crucial traits and experiences that may be influencing poor decision-making when faced with an environmental risk situation.

With respect to potential limitations of the current study, we note that the internal consistency for the CCWS was “questionable” in our sample, consistent with the study of Phillips et al., 2018 (unpublished) that reported a Cronbach’s alpha of 0.22 for the short form version of this measure (Johnson and Swedlow, 2020). The question of individual vs. country level differences has been raised, with van der Linden (2015) suggesting that using a measure designed to detect cultural leanings may be better suited to broader scale demographic purposes than to observe its effect on individuals. It is also possible that the issue lies with the Likert scale used to engage participants: having a middle-of-the-range response option (which was not included in the original measure) allowed for less commitment to either end of each axis. The effect of hierarchical cultural worldviews may, in certain cultures, be of crucial importance, as the partisan political treatment of environmental threat may be a cause of certain risk-taking behaviors (Kahan et al., 2011), particularly as those with increased hierarchical worldviews would be more likely to defer to, or expect to be deferred to as, an authority in an involuntary risk situation.

A further limitation of the study are the fit indices of the binomial regression models. While we found significance with the variables and factors distilled from our measures, low fit indices indicate the model is not as robust as we would have hoped. Given the exploratory nature of the study and the breadth of variables assessed, further research may find that there is a better battery or collection of items that may more accurately represent the factors involved. Due to funding limitations and concerns around response fatigue/participant non-completion, we were unable to include measures of global cognitive function such as Raven’s Progressive Matrices, which may have allowed us to assess and control for the effect of cognitive function on risk-taking behavior (Raven, 2000; Porter et al., 2004). Decision-making behavior is limited by and influenced by time available, in combination with cognitive load capacity; however, this was beyond the scope of our current study. Future research would do well to include such assessments (Kahneman, 2003). Locus of control (i.e., the amount of control an individual perceives he or she has over his/her life; Galvin et al., 2018) may be a suitable target for future study, given its potential relevance in risky decision-making in relation to the involuntary risk of natural hazards. As discussed earlier, the majority of research into risk-taking is focused toward understanding risk-taking through the lens of voluntary or proxy-voluntary risks. Burmudez (1999) reported that personality traits can be clustered onto factors, with agreeableness and neuroticism loading onto an “emotional character” construct that includes anxiety, depression and impatience (with an external locus of control), and conscientiousness, extraversion and openness loading on an energetic-motivational factor that includes the constructs of competitiveness, optimism, self-efficacy, and an internal locus of control. While optimism and self-efficacy may be powerfully beneficial in voluntary risk situations, an excess of self-efficacy and optimism may be contributing to undesirable outcomes in involuntary risk-taking situations. This is not to say that the opposite is better – heightened emotional coping mechanisms may result in risk-taking to avoid dealing with the emotions engendered by the situation. Establishing the extent to which locus of control impacts other mental health and demographic factors would be beneficial.

Males have been reported to be largely more risk-prone than females (Harris and Jenkins, 2006), so the consistency in this finding is not particularly surprising. As discussed, increased stress is associated with greater risk taking (Lazarus, 2000). Previous floodwater driving research has investigated the frequency with which males would engage in this risk-taking behavior. However, our study has placed focus on stress and (lack of) openness as potential reasons *why* this may occur in involuntary situations. We have also queried the preferences of the general public with regard to such behavior rather than eliciting responses from those with experience of having done so before. With some of the psychological determinants of this risk situation identified, future research would benefit from ascertaining whether there are more psychological differences between voluntary and involuntary risk, and if there are, how they could be addressed to reduce the number of individuals making dangerous decisions. We found little support for our hypotheses regarding the effects of individual personality traits on risk-taking – a somewhat surprising result given the amount of literature that indicates that these relationships should have been apparent in our results (e.g., Soane and Chmiel, 2005; Bowen et al., 2020). However, when trait effects were combined with cultural worldview, age and gender, predictive ability achieved significance, suggesting that risk-aversion is influenced by how “progressive” or “insightful” the individual’s nature. Taken together, these findings point to the need for hazard-reduction messaging that targets individuals on differing levels to account for psychosocial differences that may impact message acceptance.

## Data availability statement

The raw data supporting the conclusions of this article will be made available by the authors, without undue reservation.

## Ethics statement

The studies involving human participants were reviewed and approved by University of New England Human Research Ethics Committee. The participants provided their written informed consent to participate in this study.

## Author contributions

SB, MP, and AL contributed to conception and design of the study and wrote sections of the manuscript. SB and AL organized and cleaned the data. SB and DA performed the statistical analyses. SB wrote the first draft of the manuscript. All authors contributed to the manuscript revision, read, and approved the submitted version.

## Conflict of interest

The authors declare that the research was conducted in the absence of any commercial or financial relationships that could be construed as a potential conflict of interest.

## Publisher’s note

All claims expressed in this article are solely those of the authors and do not necessarily represent those of their affiliated organizations, or those of the publisher, the editors and the reviewers. Any product that may be evaluated in this article, or claim that may be made by its manufacturer, is not guaranteed or endorsed by the publisher.
